# Chemical composition, antioxidant and antimicrobial potential of essential oils from different parts of *Daphne mucronata* Royle

**DOI:** 10.1186/s13065-018-0495-1

**Published:** 2018-12-17

**Authors:** Iqra Ashraf, Muhammad Zubair, Komal Rizwan, Nasir Rasool, Muhammad Jamil, Shakeel Ahmad Khan, Rasool Bakhsh Tareen, Viqar Uddin Ahmad, Abid Mahmood, Muhammad Riaz, M. Zia-Ul-Haq, Hawa ZE Jaafar

**Affiliations:** 1 Department of Chemistry, Government College University, Faisalabad, 38000 Pakistan; 2 Department of Chemistry, Government College Women University, Faisalabad, Pakistan; 3 Department of Chemistry, City University of Hong Kong, 83 Tat Chee Avenue, Kowloon, China; 4 Department of Botany, University of Balochistan, Quetta, Pakistan; 5 HEJ Research Institute of Chemistry, International Centre for Chemical and Biological Sciences, University of Karachi, Karachi, Pakistan; 6 Department of Environmental Sciences and Engineering, Government College University, Faisalabad, 38000 Pakistan; 7 Department of Chemistry, University of Sargodha, Sargodha, Pakistan; 8 ORIC, Lahore College for Women University, Jail Road, Lahore, Pakistan; 9 Department of Crop Science, Faculty of Agriculture, Universiti Putra Malaysia, 43400 Serdang, Selangor Malaysia

**Keywords:** *D. mucronata*, Essential oil, Antioxidant, Leaves, Camphor

## Abstract

This research work was executed to determine chemical composition, anti-oxidant and anti-microbial potential of the essential oils extracted from the leaves and stem of *Daphne mucronata* Royle. From leaves and stem oils fifty-one different constituents were identified through GC/MS examination. The antioxidant potential evaluated through DPPH free radical scavenging activity and %-inhibition of peroxidation in linoleic acid system. The stem’s essential oil showed the good antioxidant activity as compared to leaves essential oil. Results of Antimicrobial activity revealed that both stem and leaves oils showed strong activity against *Candida albicans* with large inhibition zone (22.2 ± 0.01, 18.9 ± 0.20 mm) and lowest MIC values (0.98 ± 0.005, 2.44 ± 0.002 mg/mL) respectively. Leaves essential was also active against *Escherichia coli* with inhibition zone of 8.88 ± 0.01 mm and MIC values of 11.2 ± 0.40 mg/mL. These results suggested that the plant’s essential oils would be a potential cradle for the natural product based antimicrobial as well as antioxidant agents.
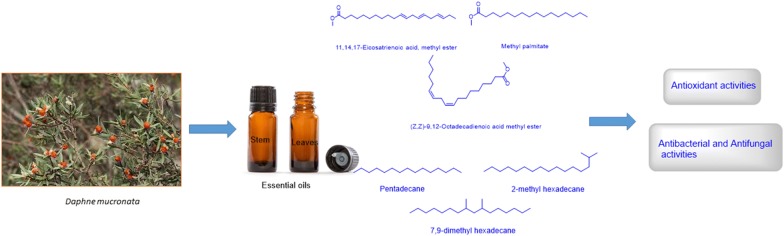

## Background

Medicinal plants are well-known since beginning of human civilization for welfare of mankind and they dwell an imperative place in the socio-cultural as well as in the health-system of indigenous communities of Pakistan. Plant’s essential oils are worthwhile natural-products that are employed as raw materials in various fields, such as cosmetics, fragrances, phyto-therapy, nutrition and spices. *Daphne mucronata* Royle belongs to the family Thymelaeaceae. Common names of this plant include Kutilal, Nirko, Laighonai (laighuanay), Kheweshk. Leaves of this plant are poisonous and applied as insect repulsive abscesses for sore and glue is used for muscular and nerve troubles [1]. Plant poultice is applied for rheumatism and sweeping [2]. The plant has attractive flowers and can be used as decorative plant [3]. The roots and shoots of *D. mucronata* Royle are considered as anthelmintic and employed in treatment of gonorrhea [4]. Fruits are multipurpose so they are used for eating purposes and for treating eye problems, to cure skin, considered as remedy for face freckles, for killing lices, ticks and are also involved in coloring leather [4, 5]. Wood is used as firewood and used in preparation of gun powder charcoal [6]. The bark is used in turmoil of bone for washing hairs and in folk medicines. Previous study revealed the presence of several phytochemicals, in this specie [7]. To date, there are no previous reports related to Phyto-chemical composition as well as biological potential of plant *Daphne mucronata* Royle essential oils. As part of our efforts [8–12] this study is, therefore, reporting for the first time the aerial parts (stem and leaves) essential oil composition, and there biological potential.

## Results and discussion

### Percentage yield and chemical composition of essential oils

**Fig. 1:**
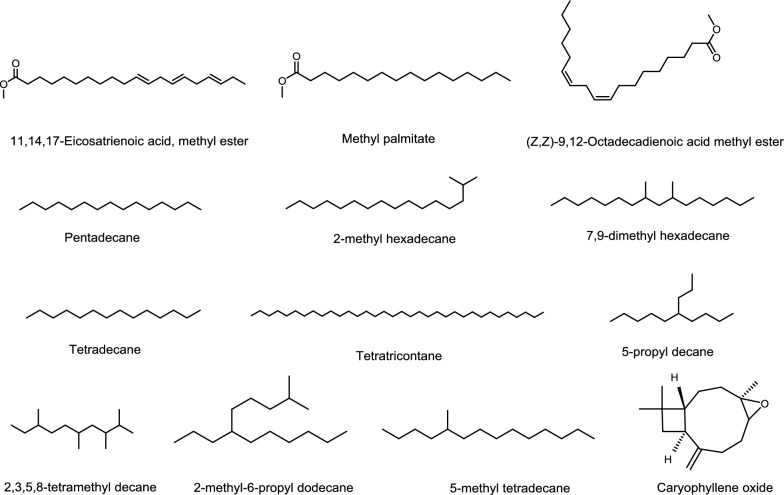
Most abundant compounds identified in *D. mucronata* (stem and leaves) essential oils

**Fig. 2:**
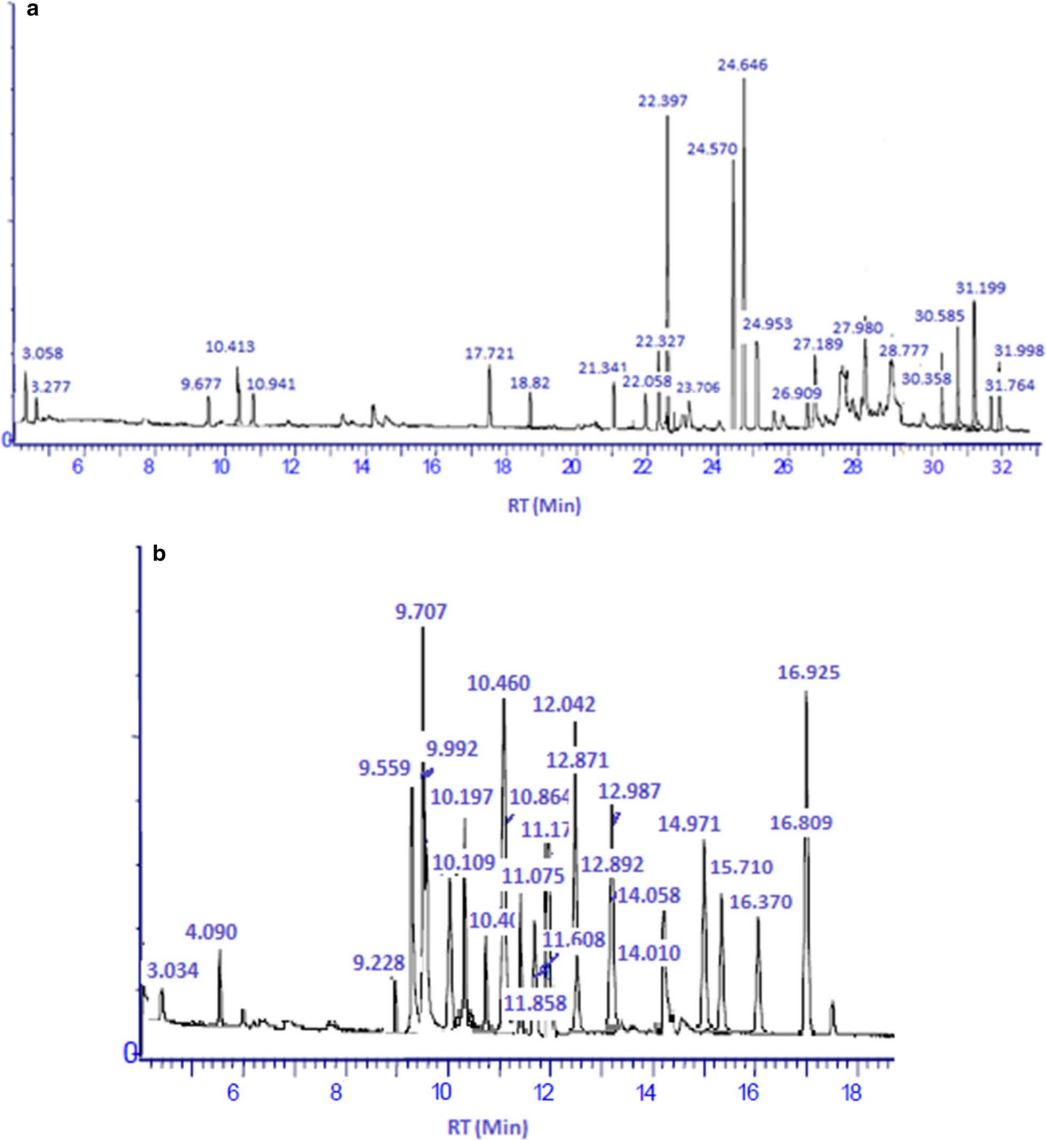
GC/MS spectra of *D. mucronata* stem (**a**) and leaves (**b**) essential oils

The yield of the essential oils (Dry plant samples) obtained from the hydrodistillation of the *D. mucronata* leaves and stem were 5.6% and 9.5% g/100 g respectively shown in Table 2. The components were identified in the essential oils with their percentage composition, relative retention time and retention indices (Table 1, Fig. 2). Twenty-seven (27) constituents were identified and quantified in the oil of *D. mucronata* leaves, representing 97.25% of the total oil. The major components were pentadecane (12.75%), 2-methyl hexadecane (8.90%), 7,9-dimethyl hexadecane (8.90%), tetradecane (7.32%), 5-Propyl decane (6.16%), 2,3,5,8 tetramethyl hexadecane (5.81%), 2-methyl-6-propyl dodecane (5.11%), 5-methyl tetradecane (5.10%) (Table 1, Fig. 1). In the oil of *D. mucronata* stem twenty-seven constituents (91.2%) were identified. The major compounds were 11,14,17-eicosatrienoic acid, methyl ester (18.57%), methyl palmitate (16.0%), (Z,Z)-9,12-octadecadienoic acid methyl ester (13.99%), tetratriacontane (6.65%), caryophyllene oxide (5.94) (Table 1, Fig. 1). GC/MS spectra of both (stem and leaves) essential oils are presented in Fig. 2. The essential oils consisted of some straight chain alkanes, fatty acids, methyl esters and aromatics, which may be involved in antioxidant and antimicrobial activities.

**Table 1:** GC/MS analysis of *D. mucronata* essential oils

Retention indices	Compound name	% Area	
		Leaves	Stem
716	Cyclohexyl methane	–	0.96
805	*trans*-1,2-dimethylcyclohexane	0.86	–
820	2,2,3,4-Tetramethylpentane	2.08	3.47
944	2,3,3-Trimethyl-octane	1.24	–
970	5-(1-methylpropyl)-nonane	3.13	–
1044	Camphor	–	1.27
1099	2,2-dimethyl octanol	1.26	–
1114	3-Thujanone	–	0.6
1138	*trans*-5,6-Epoxydecane	0.84	–
1175	1-Terpinen-4ol	–	0.31
1264	2-Methyl-6-propyl dodecane	5.11	–
1298	2,3,5,8-Tetramethyl decane	5.81	0.37
1322	7,9-dimethyl hexadecane	8.90	–
1399	Tetradecane	7.32	–
1445	2-Bromo dodecane	1.20	–
1454	5-Methyl tetradecane	5.10	–
1500	Pentadecane	12.75	–
1542	7-Methyl pentadecane	1.63	–
1563	Caryophyllene oxide	–	5.94
1660	2,6,10,15-Tetramethyl heptadecane	2.71	–
1664	Ar-tumerone	–	3.94
1666	2-Methyl hexadecane	8.90	–
1686	(*Z*)-11-Pentadecenal	2.88	–
1719	8-Hexyl pentadecane	–	0.86
1745	8-Methyl heptadecane	–	0.34
1800	5-Propyl decane	6.16	–
1848	Hexahydrofarnesyl acetone	–	2.35
1854	5-Methyl octadecane	1.30	–
1878	Methyl palmitate	–	16.02
1897	7-Hexadecenoic acid, methyl ester, (Z)-	–	0.31
1922	Dibutyl phthalate	0.86	–
1974	Methyl isoheptadecanoate	–	0.35
1984	*n*-hexadecanoic acid	1.74	–
1999	d-Mannitol, 1-decylsulfonyl-	2.89	–
2000	Eicosane	2.66	–
2067	(Z,Z)-9,12-octadecadienoic acid methyl ester	–	13.99
2100	Heneicosane	–	1.50
2116	11,14,17-Eicosatrienoic acid, methyl ester	–	18.57
2167	Decane, 1,1′-oxybis-	2.52	–
2190	Octadecanoic acid, methyl ester	–	2.36
2327	Eicosanoic acid, methyl ester	–	0.91
2400	Tetracosane	–	0.42
2413	Octadecane,3-ethyl-5-(2-ethylbutyl)-	1.83	–
2525	1,2- diisooctyl benzenedicarboxylic acid ester	4.76	2.12
2527	Behenic acid, methyl ester	–	1.40
2714	Tetracosanoic acid, methy ester	–	1.44
2790	*trans*-Squalene	–	2.43
2908	Hexacosanoic acid, methyl ester	–	0.95
3132	Tocopheryl acetate	0.81	–
3400	Tetratriacontane	–	6.65
3600	Hexatriacontane	–	1.16

### Antioxidant and antimicrobial potential of essential oils

Free radicals are highly reactive species which are produced in human body due to various reactions taking place in human body, radiations exposure and environment pollution. These radicals are responsible for damaging human health and cause many diseases. Antioxidants are responsible for scavenging the radicals and convert them to less reactive species. Plants are best natural source of antioxidants. Antioxidant potential of plant *D. mucronata* essential oils was investigated by DPPH scavenging assay and by measuring % Inhibition of peroxidation in linoleic acid system. The plant oils showed moderate antioxidant activity (Table 2). Stem essential oil proved most active, with an IC_50_ value of 45.46 ± 0.04 µg/mL, followed by leaves essential oil (IC_50_ = 85.15 ± 0.31 µg/mL). Maximum  % inhibition of peroxidation in linoleic acid system was showed by the stem essential oil (64.16 ± 0.93) followed by leaves essential oil (37.57 ± 0.89). So stem essential oil showed maximum antioxidant potential as compared to leaves of plant. When the results of DPPH scavenging activity (IC_50_) and the percent inhibition of peroxidation in linoleic acid system were compared with standard BHT (Butylated hydroxytoluene), both essential oils showed significantly (*p *< 0.05) less activity.

**Table 2:** % Yield and antioxidant analysis of *D. mucronata* Royle essential oils

Samples, standard compound	% Yield g/100 g	% Inhibition of peroxidation in linoleic acid	DPPH radical scavenging IC_50_ (µg/mL)
Leaves essential oil	5.6±0.005	37.57 ± 0.89	85.15 ± 0.31
Stem essential oil	9.5±0.008	64.16 ± 0.93	45.46 ± 0.04
BHT	–	89.1 ± 0.78	9.01 ± 0.10

Values are mean ± SD of three separate experiments (P < 0.05) BHT (butylated hydroxytoluene)

**Fig. 3:**
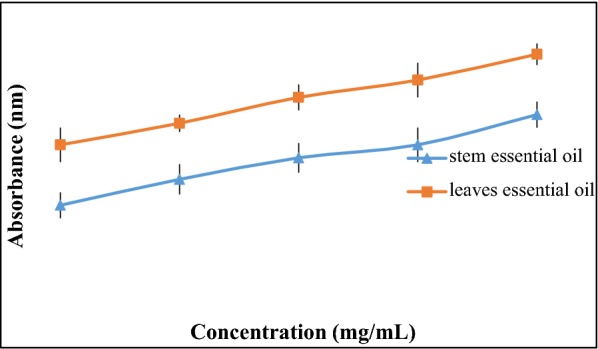
Reducing potential of *D. mucronata* Royle essential oils

The reducing potential of plant essential oil (stem, leaves) was investigated at different concentrations (2.5–10 mg/mL). The plant (stem, leaves) essential oils satisfied the test of reducing power by giving a linear increase to absorbance with concentration. Leaves essential oil showed maximum reducing power (Fig. 3).

Micro-organisms are responsible for causing damage to human health, spoilage of food and many other problems. Micro-organisms have become drug resistant, so there is need to discover new sources against disease causing micro-organisms. Essential oils and their constituents play key role in inhibiting growth of micro-organisms [13]. The antimicrobial potential of *D. mucronata* essential oils was determined against various pathogens (Table 3). The results indicated that the stem essential oil sowed potent inhibitory activity against only *C. albicans*, with the highest inhibition zone (22.2 ± 0.01 mm) and the lowest MIC value (0.98 ± 0.005 mg/mL). Leaves essential oil was active only against *C. albicans* and *E. coli*. Growth of *C. albicans* was strongly inhibited with large inhibition zone (18.9 ± 0.20 mm) followed by MIC value (2.44 ± 0.002 mg/mL). Leaves essential oil showed moderate activity against *E. coli* (zone of inhibition = 8.88 ± 0.01 mm; MIC = 11.2 ± 0.40). Both essential oils were inactive against *Staphylococcus aureus*, *Nitrospira moscoviensis*, *Bacillus cereus*, *Staphylococcus epidermidis*, *Aspergillus flavus* and *Aspergillus niger* (Table 3). These strains were resistant to *D. mucronata* Royle essential oils. The results of antimicrobial activity were compared to standard drugs Rifampicin and fungone for bacterial and fungal strains respectively. Antimicrobial activity of the some species of *Daphne* has already been documented in literature [14, 15]. Mikaeili and co-workers [16] reported the anticandidal activity of 1,2-benzenedicarboxylic acid, diisooctyl ester as this compound was present in both stem and leaves essential oil in good concentration, so essential oils showed potent antimicrobial activity against *candida albicans*. It has been suggested that the antimicrobial and antioxidant activities of essential oils is attributable to the presence of compounds such as alcohols, aldehydes, alkenes, esters and ethers [17], some of them found in the oils of *D. mucronata* (Table 1). For instance, the essential oils of *D. mucronata* contain substances as, 3-Thujanone, camphor, Caryophyllene oxide, trans-1,2-dimethylcyclohexane, tetradecane, hexahydrofarnesyl acetone, 5-methyl octadecane found in several vegetal species, which have demonstrated various pharmacological effects [18–21]. It is possible that the antimicrobial and antioxidant activities demonstrated by the essential oils extracted from *D. mucronata* could be attributed to these components. These results are very promising as the oils can be used as a good source of antioxidant and antimicrobial compounds.

**Table 3:** Antimicrobial activity of *D. mucronata* Royle essential oils

Tested microbes	Leaves essential oil		Stem essential oil		Standard drugs	
	Zone of inhibition (mm)	MIC mg/mL	Zone of inhibition (mm)	MIC mg/mL	Zone of inhibition (mm)	MIC (mg/mL)
*A. flavus*	–	–	–	–	19.0 ± 0.60	0.86 ± 0.001
*A. niger*	–	–	–	–	20.7 ± 0.55	0.48 ± 0.001
*B. cereus*	–	–	–	–	21.7 ± 0.49	0.97 ± 0.0003
*C. albicans*	18.9 ± 0.20	2.44 ± 0.002	22.2 ± 0.01	0.98 ± 0.005	23.8 ± 0.67	0.25 ± 0.0001
*E. coli*	8.88 ± 0.01	11.2 ± 0.40	–	–	25.26 ± 0.3	0.46 ± 0.0002
*N. moscoviensis*	–	–	–	–	22.9 ± 0.43	0.39 ± 0.0007
*S. aureus*	–	–	–	–	30.0 ± 0.32	0.25 ± 0.0001
*S. epidermidis*	–	–	–	–	23.4 ± 0.50	0.33 ± 0.0003

Values are mean ± S.D of three separate experiments (P < 0.05)

Rifampicin and fungone were used as standards for bacterial and fungal strains respectively

## Materials and methods

### Plant materials

The entire plant “*D. mucronata* Royle” was attained from Quetta, Pakistan. The plant was identified by Prof. Dr. Rasool Bakhsh Tareen, Botany Department, University of Balochistan, Quetta, Pakistan, where we deposited sample-specimen (Voucher # DM-RBT-09).

### Essential oil extraction

For the essential oils extraction, 50 g of each part (stem and leaves) of powdered plant materials dried under the shady place, were hydro distillated by employing a Clevenger-type device for 5 h. Sodium sulphate (Na_2_SO_4_) was used for drying the extracted essential oils, then after filtration oils were stored in a vial at 4 °C till start of further analysis.

### GC–MS analysis

The GC–MS examinations of the essential-oils were done by employing a GCMS-QP2010 (SHIMADZU, Japan). The conditions for GC–MS examinations of essential-oils were: the sample-solution (1 µL/mg) inserted in split-less mode via manually and the time for sampling was 1 min. Then the temperature 200 °C was established for the injection port. The gas chromatography was fitted out with the column of DB-5 capillary whose internal diameter, length and film thickness were 0.25 mm, 30 m and 0.25 µm respectively. A three step gradient temperature was accomplished for oven: accordingly, 45 °C for 5 min was set as an initial temperature. Then, initial temperature was upraised at a rate of 10 °C upsurge per min up to 150 °C, trailed by 5 °C per min upsurge up to 280 °C and finally, temperature touched to the 325 °C at 15 °C per min upsurge and keep it for five min. At that time, the Helium was employed at a flow-rate of 1.1 mL per min (liner velocity and pressure were 38.2 cm/sec and 60 kPa respectively). In a scanning mode, the fragments/ions were scrutinized over 40–550 *m/z*. The components were identified and recognized on the bases of their mass spectra comparison with the NIST mass spectral library [22, 23]. Retention indices was calculated by following given formula:

$${\text{Retention indices }}\left( {\text{RI}} \right)\, = \, 100{\text{ C}}_{\text{n}} \, + \, 100 \, \left( {{\text{C}}_{{{\text{n}} + {\text{i}}}} - {\text{C}}_{\text{n}} } \right) \, \times {\text{ T}}_{{{\text{R}}({\text{x}})}} - {\text{T}}_{{{\text{R}}({\text{n}})}} \div {\text{ T}}_{{{\text{R}}({\text{n}} + {\text{i}})}} - {\text{T}}_{{{\text{R}}({\text{n}})}}$$

C_n_ and C_n+i_ represents carbon numbers of carbon standards eluting before and after compounds to be identified.

T_R(x)_ = represents retention time of compounds to be identified

T_R(n)_ = retention time of carbon (C_n_)

T_R(n+i)_ = retention times of carbon (C_n+i_)

### Antioxidant activity

#### DPPH radical scavenging assay

The antioxidant propensity of plant essential oils was checked by measuring their ability to scavenge stable DPPH free radical following the standard protocol as reported earlier by Rizwan and co-workers [24] with slight modifications. The 1 mL of 90 μM DPPH solution was mixed with the samples (from 10 to 500 μg mL^−1^) and 95% methanol was used to made the final volume up to 4 mL. The Butylated hydroxyl-toluene (BHT) was served as an external standard. Then the sample incubation was done for 1 h at the temperature of (25 °C). After that, the absorbance was examined at 515 nm. By the following formula Percent DPPH radical scavenging was calculated:

$${\text{Radical scavenging }}\left( \% \right)\, = \, 100 \, \times \, \left( {{\text{A}}_{\text{blank}} - {\text{A}}_{\text{sample}} /{\text{A}}_{\text{blank}} } \right)$$

where A_blank_ is the absorbance of the control (containing all reagents except the test samples), and A_sample_ is the absorbance of the test samples. IC_50_ values, which represented the concentration of samples that caused 50% scavenging, were calculated from the plot of inhibition percentage against concentration.

#### Percentage-inhibition of linoleic peroxidation

Antioxidant potential of *D. mucronata* essential oils was evaluated by measuring percent-inhibition of linoleic peroxidation [12]. The 5 mg of plant’s essential oil sample mingled with the 0.13 mL linoleic acid solution, 10 mL of 0.2 M sodium phosphate buffer of pH ~ 7, 10 mL of 99.8% ethanol, and diluted with distilled water (up to 25 mL). Then the resultant reaction mixture was hatched at 40 °C for 360 h (15 days) and extent of oxidation was examined [15]. After that, sample solution (0.2 mL), ferrous chloride solution (0.2 mL) (20 mM in 3.5% HCl w/v), 75% ethanol (10 mL), and 30% ammonium thiocyanate (0.2 mL) were mixed together consecutively. Finally, the absorbance of reaction mixture was noted at 500 nm after stirring for 3 min. Experiment was also performed on control, which consist only on linoleic acid without sample. As a positive control, the BHT was employed. By a following equation, Percent-inhibition of linoleic acid peroxidation was determined:

$$\% {\text{ Inhibition}}\, = \, 100{-}\left[ {\left( {{\text{Abs}}.{\text{ increase of sample at 36}}0 {\text{h}}/{\text{Abs}}.{\text{ increase of control at 36}}0 {\text{h}}} \right)\, \times \, 100} \right]$$

#### Analysis of reducing power

At different concentrations (2.5–10 mg), the plant oils were mingled with 1% potassium ferricyanide (5 mL) and 5 mL of sodium phosphate buffer (0.2 M, pH 6.6) solution. For 20 min at 50 °C, the reaction mixture was heated and after that, 10% of trichloroacetic acid (5 mL) was mixed with heated reaction mixture. Then the resultant solution was subjected for centrifugation for 10 min at 5 °C at the rate of 980 rpm. At that time, the 5 mL of upper layer of reaction mixture was dissolved in 5 mL of distilled H_2_O. As a final point, 1 mL of 0.1% freshly prepared FeCl_3_ solution was added in it. At 700 nm absorbance was noted and result were obtained in triplicates [12].

### Antimicrobial assay

#### Microbes

Four different bacteriological strains (*Bacillus cereus* ATCC 14579, *Escherichia coli* ATCC 25922, *Staphylococcus epidermidis* ATCC 12229 and *Nitrospira moscoviensis* locally isolated) and three different fungal strains (*Aspergillus niger* ATCC 10595*, Candida albicans* ATCC 10231, *Aspergillus flavus* ATCC 32612) were used to check the antimicrobial effects of essential oils. For this study, pure microbial organisms were provided by Department of Veterinary Microbiology (DVM) (University of Agriculture Faisalabad (UAF), Pakistan). The nutrient agar was employed to culture bacteriological strains overnight at 37 °C while potato dextrose agar (PDA) was cast off for the development and culturing of fungal strains at 28 °C.

#### Disc diffusion method

The antimicrobial potential of plant essential oils was determined by Disc Diffusion method [25]. For this, the 6 mm diameter discs were employed whose soaking was performed with 20 mg/mL essential oil (100 μL/disc). Moreover, soaked disk were placed on the inoculated agar. Discs without samples were used as negative control. The fungone (100 μL/disc) and Rifmapicin (100 μL/disc) were served as a positive control for fungal and bacteriological strains respectively. The incubation of petri-dishes for bacteria were performed at 37 ± 0.1 °C for 24 h while for fungi at 28 ± 0.3 °C for 48 h. For the results, zones of inhibition (ZOIs) formation were measured on the agar media.

#### Minimum inhibitory concentration (MIC)

The resazurin microtitre-plate assay was employed to determine the minimum inhibitory concentration (MICs) of the *D. mucronata* essential oils [26].

### Statistical analysis

All samples were analyzed in triplicate. Data were analyzed by analysis of variance (ANOVA) using Costat (Version 3.8) statistical software.

## Conclusions

We have investigated essential oils from aerial parts of *Daphne mucronata* obtained by hydro-distillation process. Fifty-one different compounds were found in stem and leaves essential oils by GC–MS analysis. These compounds made the essential oils very effective in antimicrobial and antioxidant potential. Our study revealed that oils obtained from *D. mucronata* could be a promising source of effective antioxidant and antimicrobial compounds and may play vital role for discovery of new drugs against pathogenic diseases. Both of these essential oils may play an important role in flavoring and cosmetic industry.
